# Effect of methylene blue on hemodynamic response in the early phase of septic shock: A case series

**DOI:** 10.1097/MD.0000000000032743

**Published:** 2023-01-27

**Authors:** Fabio Luis-Silva, Mayra Gonçalves Menegueti, Lucas Sato, Leandro Moreira Peres, Corina dos Reis Sepeda, Bruno C. Petroski-Moraes, Mariana Dermínio Donadel, Gabriela Bortoleto Gallo, Maria Cecília Jordani, Fabiola Mestriner, Christiane Becari, Anibal Basile-Filho, Paulo R. B. Evora, Olindo Assis Martins-Filho, Maria Auxiliadora-Martins

**Affiliations:** a Division of Intensive Care Medicine, Department of Surgery and Anatomy, Ribeirão Preto Medical School, University of São Paulo, Ribeirão Preto, São Paulo, Brazil; b Ribeirão Preto Nursing School, University of São Paulo, Ribeirão Preto, São Paulo, Brazil; c Division of Cardiac Surgery, Department of Surgery and Anatomy, Ribeirão Preto Medical School, University of São Paulo, Ribeirão Preto, São Paulo, Brazil; d Division of Vascular and Endovascular Surgery, Department of Surgery and Anatomy, Ribeirão Preto Medical School, University of São Paulo, Ribeirão Preto, São Paulo, Brazil; e René Rachou Institute, Oswaldo Cruz Foundation, FIOCRUZ-Minas, Belo Horizonte, Minas Gerais, Brazil.

**Keywords:** cytokines, lactate, methylene blue (MB), nitric oxide, septic shock

## Abstract

Methylene blue (MB) has been used to increase blood pressure in septic shock, acting on the activity of guanylate cyclase and nitric oxide synthase.

**Patience concerns:**

The aim of this study is to demonstrate the benefit of MB in early phase of septic shock.

Diagnoses: We report 6 cases of patients with septic shock with up to 72 hours of evolution.

**Interventions:**

We used MB after fluid replacement, use of norepinephrine and vasopressin. Patients received a loading dose of MB and maintenance for 48 hours.

**Outcomes:**

All patients presented a reduction in the dose of vasopressors and lactate levels soon after the administration of the loading dose of MB, an effect that was maintained with the maintenance dose for 48 hours. Interleukin 6 and interleukin 8 were elevated at the beginning of the septic condition, with a progressive and marked reduction after the beginning of MB infusion, demonstrating a role of MB in reducing the inflammatory activity.

**Lessons:**

This case series suggests that MB used early in the treatment of septic shock may be useful in reducing vasopressor dose and lactate levels. Further studies are still required to further validate these findings.

**Key points**

Methylene blue (MB) has been used to increase blood pressure in septic shock, acting on the activity of guanylate cyclase and nitric oxide synthase.
MB used early in the treatment of septic shock may be useful in reducing vasopressor dose, inflammatory cytokines and lactate levels.
MB used in early phase of septic shock is more useful than in late rescue treatment.

## 1. Introduction

Organ dysfunction characterizes septic shock, secondary to an exacerbated response to infection.^[1]^ Mortality varies between 38 and 46.5%,^[2]^ however we must consider the possibility of undersized data because of failures in the documentation of data from developing countries. Population-level epidemiological data for sepsis are scarce and virtually non-existent for low- and middle-income countries, and a provisional extrapolation of data from high-income countries suggesting global estimates of 5.3 million deaths annually.^[3]^ Such data may vary mainly because of the diversity in the diagnostic criteria, as well as in the different treatment adopted in the countries.^[1]^ The diagnosis of septic shock in patients with a presumed or confirmed focus of infection associated with mean arterial pressure (MAP) ≤ 65 mm Hg and lactate ≥ 2.0 mmol/L after adequate fluid resuscitation. In this phase, different abnormalities in the most diverse systems can occur, such as cellular, metabolic, endocrine, pulmonary, renal, hematological, and neurological abnormalities.^[4]^

The septic shock treatment protocol involves intravenous fluid replacement, infusion of vasopressors and administration of antibiotics within the first hour.^[1,3]^ Introducing this triad as a standardized treatment systematized the approach, but mortality still remains high, especially in underdeveloped countries. In addition to fluid replacement and the use of vasopressors and antibiotics, the use of low-dose corticosteroids is also indicated in cases of shock that do not initially respond to vasopressors. Therefore, it is crucial to study new drugs that could help maintain hemodynamic stability until the antibiotic can act and fight the infectious focus. In this direction, we highlight the use of methylene blue (MB), an inhibitor of the nitric oxide (NO) pathway, as an adjuvant in the reversal of vasodilation that occurs as the initial mechanism of septic shock.^[5]^

There are few studies in the literature on the use of MB in septic shock, with different protocols, and that evaluate both the vasopressor sparing effect and the reduction of inflammatory cytokines, but with small populations and with inconclusive results.^[6,7]^

MB becomes interesting because it is a drug used since the 19th century with proven hemodynamic effects since 1976^[8]^ besides presenting safety in its use, as it has minimal side effects when used in adequate doses.

MB is a phenothiazine-related heterocyclic aromatic molecule, responsible for reducing cyclic guanosine monophosphate (cGMP) levels, reducing the vasodilatory effect via binding of the heme iron portion of soluble guanylyl cyclase (sGC), blocking the action of sGC in smooth muscle vessels.^[9]^ In addition, MB can inhibit inducible NO synthase and clear NO, reducing its vasodilatory effects approximately 30 to 60 minutes after intravenous administration. Its excretion occurs via the biliary, fecal and renal routes, and intravenous administration has a plasma half-life of 5 to 6 hours,^[10]^ therefore continuous infusion may be beneficial after administration of a loading dose for 48 hours.^[11]^

Because of this mechanism of action by selective blockade of sGC and inhibition of NO synthase, MB is a selective agent of microcirculation dysregulation in cases of upregulation of NO, and may be an option to treat refractory shock unresponsive to conventional treatment.^[11]^ Therefore, MB is a potential new tool in the treatment of septic shock, associated with standard care. Because of the shorter duration of action (2–4 hours), prolonged or continuous infusions may be necessary in patients with persistent hypotension,^[10]^ however the use in the early phase of septic shock is perhaps more sensible and not as a late rescue treatment,^[8]^ since tissue hypoxia with a high degree of anaerobic metabolism was associated with loss of hemodynamic responsiveness to its administration.^[12]^

Our hypothesis is that MB may contribute to reduce the infusion of vasopressors, reverse refractory vasodilation, improve tissue perfusion, and reduce inflammatory mediators. Delaying mitochondrial death induced by NO, besides being a low-cost drug, few side effects and easily accessible in all health units.

**Figure 1.**
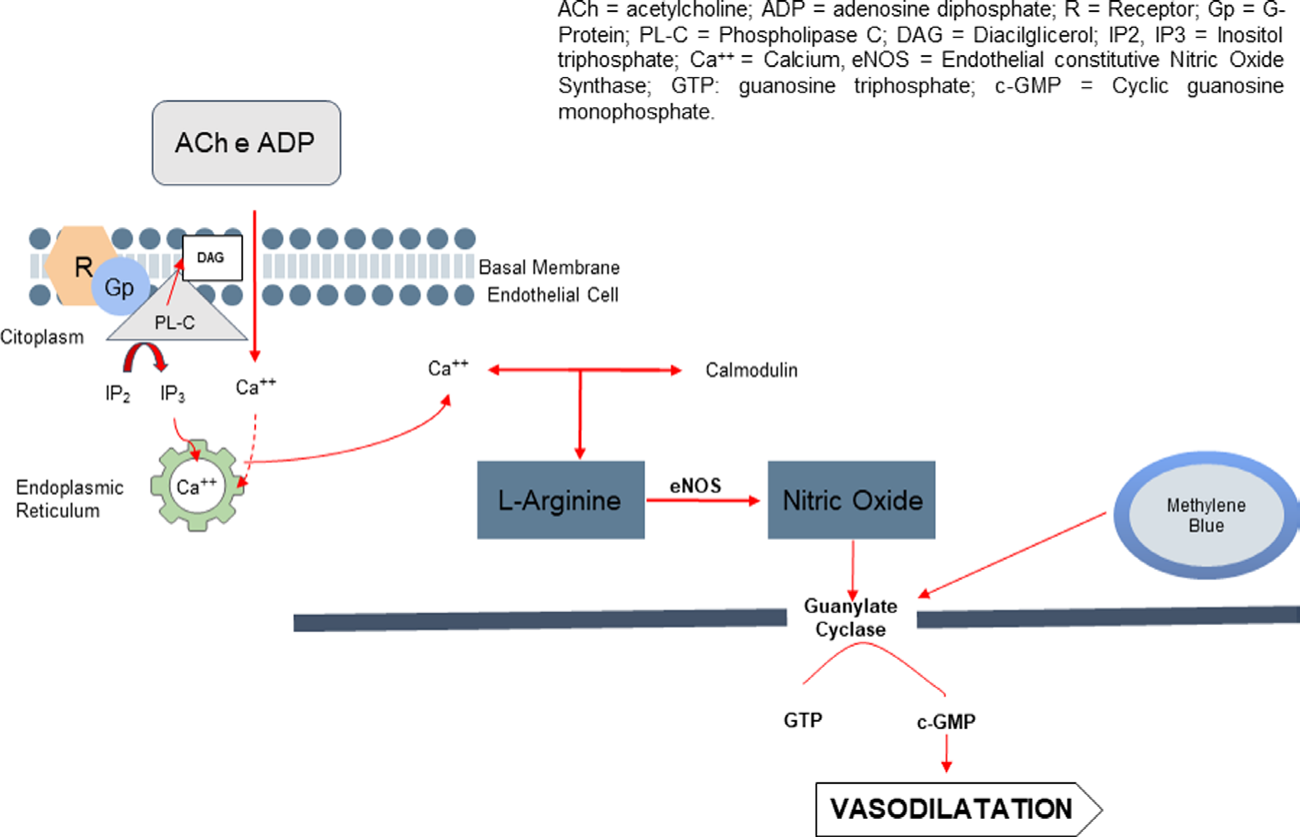
The mechanism of methylene blue action.

Figure 1 represents the main action mechanisms of MB. Noting that high levels of NO and activation of GCs are the main reasons for vasodilation. By inhibiting guanylate cyclase, MB acts to reduce vasodilation.

This case series aims to report 6 cases of patients treated with standard therapy associated with MB in the first 72 hours of septic shock onset using a protocol predefined in the literature.^[13]^

### 1.1. Material and methods

This is a case series conducted in an intensive care unit (ICU) of a tertiary university hospital in Brazil. All the legal guardians of the included patients agreed and signed the free and informed consent form, and we could carry the study out. The Ethics Committee of the Hospital das Clínicas of the Faculty of Medicine of Ribeirão Preto approved this protocol–number: 562/2017, version: 2/2016.

Inclusion criteria were: patients between 18 and 80 years of age, of all sexes, <72 hours after the diagnosis of septic shock performed according to the definitions of Sepsis 3.^[14]^ All patients underwent lactated Ringer’s infusion and were using noradrenaline at a dose ≥ 0.2 µg/kg/min and vasopressin at the maximum dose for septic shock (0.04 IU/min).

Exclusion criteria were: pregnancy or puerperium, previous septic shock during the same hospitalization, patients in severe immunosuppression (neutrophils < 500 mm3), use of serotonergic drugs, linezolid or monoamine oxidase inhibitors that may interfere with the pharmacodynamics of MB, palliative care, imminent death and patients deprived of liberty.

All patients underwent invasive hemodynamic monitoring using the Edwards Lifesciences Corporation EV 1000®, USA, platform with VolumeView® and PreSep® catheters. Vasopressor doses and serum lactate levels were recorded in the pre-infusion period of MB (Time [T] 1), after 20 minutes (T2), 2 hours (T3), 24 hours (T4) 48 hours after the start of the infusion (T5), and 24 hours after the suspension of MB (T6), to assess the hemodynamic behavior. Was applied the Simplified Acute Physiology Score^[15]^ on admission and the Sequential Organ Failure Assessment score^[16]^ diary during the first 4 days of hospitalization. All patients underwent mechanical ventilation with a microprocessor-based mechanical ventilator (EVITA XL®, Drägermedical, Germany).

**Figure 2.**
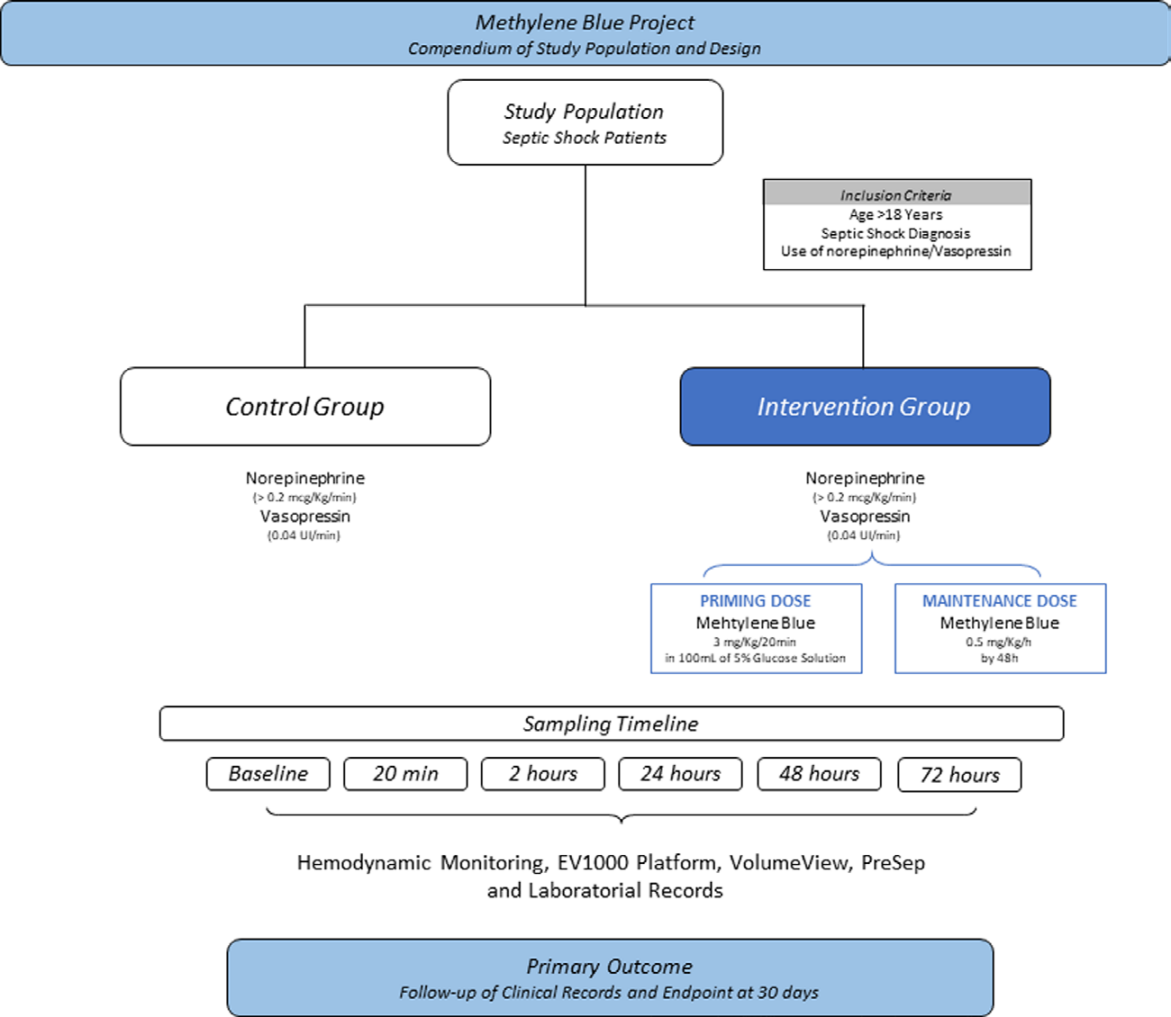
Compendium of study population and design.

All patients received progressive doses of noradrenaline and vasopressin, until hemodynamic stabilization, associated with MB at a loading dose of 3mg/kg for 20 minutes and maintenance of 0.5 mg/kg/h for 48 hours according to the study protocol (Fig. 2). Besides to the hemodynamic study, plasma determination of serum nitrate (NO3) and inflammatory cytokines was performed in parallel with 5 of the 6 patients. To perform these analyses, the samples were collected and stored in a freezer at −70 ºC.

For the determination of NO, the samples were deproteinized by incubation with 95% ethanol for 30 minutes and centrifuged at 10,000 rpm for 5 minutes. With the supernatant got, the NO/ozone chemiluminescence assay was performed. The sample (5 µL) was injected into the reaction chamber containing the reducing agent (0.8% vanadium chloride in 1N HCl) at 80 °C, which converted NO3 into NO in equimolar amounts. Nitrogen gas extracted NO into the chemiluminescence chamber of the Sievers NO Analyzer (Sievers 280i NOA, Sievers, Boulder, CO, EUA) where it reacts with ozone, emitting red light: NO + O_3_→ NO_2_ + O_2_; NO_2_ → NO_2_ + hv.

The photon emitted by the reaction is detected and converted into an electrical signal and analyzed in the computer. Area under the curve generated by the electric current corresponds to the concentration of NO in the sample. The NO3 concentration was calculated using a curve using sodium NO3 (100 a 1µM) as default. The results were expressed in µM13.

Plasma determination of interleukin (IL)-6, IL-8, IL-10 and tumor necrosis factor (TNF-α) was performed at all times studied to characterize the inflammatory response in septic shock and the action of MB in this response. Cytokines were quantified by the enzyme-linked immunosorbent assay method and the protocol was performed according to the kit instructions B&D Systems and R&D Systems, USA. Optical density values were read on a microplate reader VERSAmax, USA (Molecular Devices) at 450nm and calculated from a standard curve (4-PL) by the software SoftMax Pro v5 Molecular (Devices, LLC, Attn: Legal Department, 3860 N. First St., San Jose, CA 95134). The results were expressed in pg/mL.

## 2. Results

Six critically ill patients admitted to the ICU were included, with a mean SAPS-3 of 82 and a mean risk of death of 84%, diagnosed with septic shock within the first 72 hours of evolution (Table 1). The demographic, clinical, and microbiological characteristics of the patients are shown in Table 1. All patients received MB treatment according to the study protocol and the standard treatment was instituted according to the decisions of the intensivist according to the institutional protocol that advocates infusion of fluids, use of vasopressors (noradrenaline followed by vasopressin after a dose of 0.2 µg/kg/min of the first one) and corticosteroids when there was no response to vasopressors.

**Table 1:** Demographic, clinical and microbiological characteristics of the study population.

	Case 1	Case 2	Case 3	Case 4	Case 5	Case 6	Mean (SD)
Gender	Male	Male	Male	Male	Female	Male	-
Age (yr)	34	24	55	41	50	54	43 (11)
Comorbidity	None	Crohn disease	Alcoholism, PH	Smoking alcoholism	DVT, thrombophilia	Alcoholism	-
Infection source	Abdominal	Abdominal	Pulmonary	Pulmonary	Pulmonary	Articular	-
Identified agent	*E. coli, K. pneumoniae* and *A. baumannii*	None	None	*A. baumannii*	*A.baumannii* MDR	None	-
Culture sample	Bile	None	Tracheal secretion	Tracheal secretion	Tracheal secretion	None	-
Mechanical ventilation	Yes	Yes	Yes	Yes	Yes	Yes	-
ICU length of stay (d)	6	16	10	19	21	10	14 (5)
Dialysis	Yes	No	Yes	Yes	No	Yes	-
SOFA score (mean)*	15	4	13	14	7	12	-
SAPS 3	83	91	76	98	64	82	82 (11)
Risk of death from SAPS 3	89	94	81	96	58	88	84 (13)
Death	Yes	No	No	No	No	No	-

*A baumannii* = *Acinetobacter baumannii*, DVT = deep vein thrombosis, *E. coli* = *Escherichia coli*, ICU = intensive care unit, *K. pneumoniae* = *Klebsiella pneumoniae*, MDR = multidrug-resistance, PH = pulmonary hypertension, SAPS 3 = Simplified Acute Physiology Score 3, SD = standard deviation, SOFA = Sequential Organ failure Assessment.

* mean of the first 4 days at ICU.

Half of the patients had some microorganism identified in cultures (blood, tracheal secretion, bile and urine). In the other patients, antimicrobial therapy was instituted according to the suspected focus and microbiological characteristics of the hospital (Table 1).

In the present study, the 6 patients evolved with a reduction in vasopressors and lactate levels, soon after the administration of the loading dose of MB, an effect that was maintained with the maintenance dose for 48 hours.

**Figure 3.**
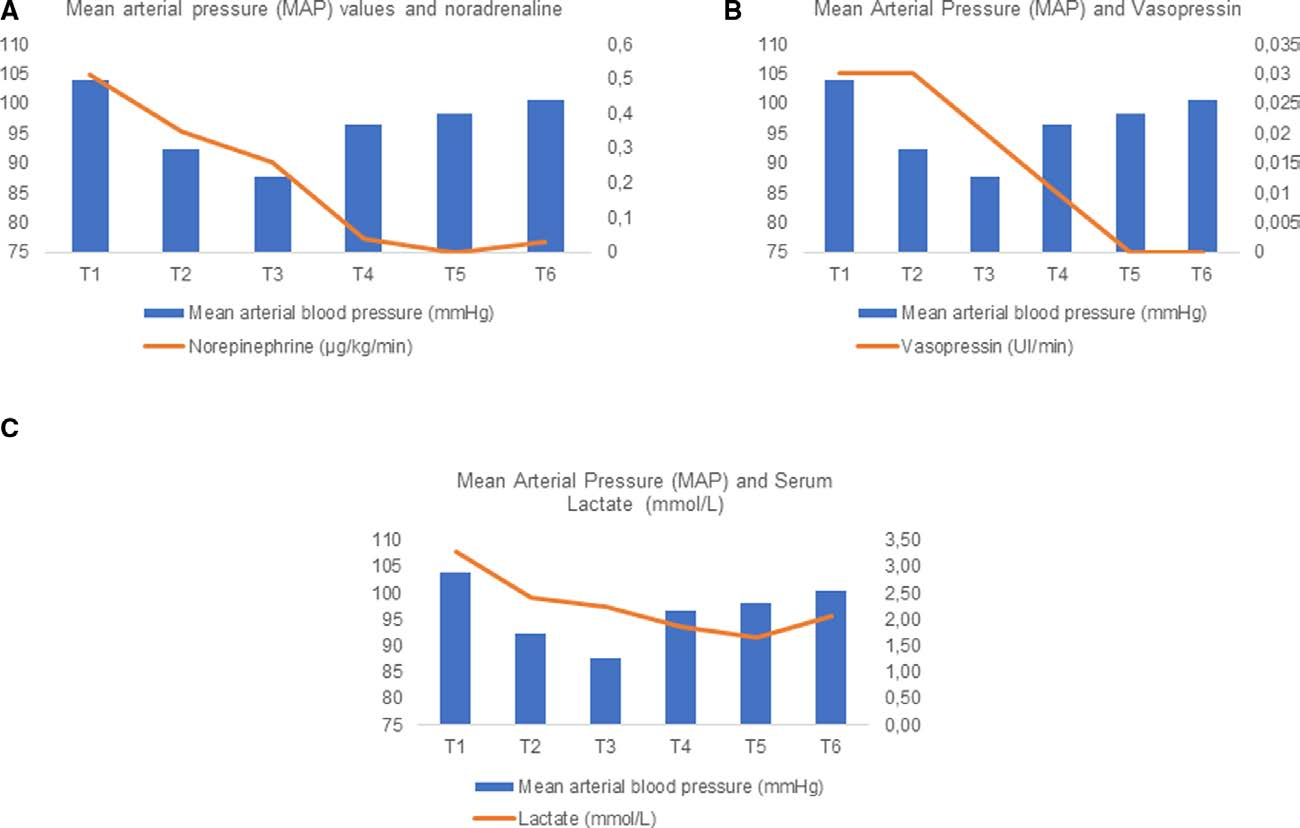
Mean arterial pressure (MAP) values and noradrenaline doses in µg/kg/min at the 6 study times. (A) There was a reduction in the noradrenaline dose shortly after the administration of the MB loading dose with maintenance for 48 hours. MAP values and vasopressin doses in U/min at the 6 study times. (B) We observed that there was a progressive reduction in the dose of vasopressin with withdrawal within 48 hours. MAP and serum lactate (mmol/L) values at the 6 study times. (C) We observed a progressive drop in serum lactate levels demonstrating a positive effect of MB on tissue perfusion. MB = methylene blue.

Mean noradrenaline doses were 0.51 µg/kg/min, 0.35 µg/kg/min, 0.26 µg/kg/min, 0.04 µg/kg/min and 0 from T1 to T5 respectively, MAP remained above 65 mm Hg, indicating the maintenance of hemodynamic stability even with progressive reduction of noradrenaline. There was a return of low doses of noradrenaline (0.03 µg/kg/min) 24 hours after the suspension of MB (T6) (Fig. 3).

When evaluating the dose of vasopressin, we observed that at T4 there was a dose reduction and at T5 the patients were no longer using this vasopressor (Fig. 3). The reduction started after 24 hours, since the institutional protocol recommended weaning initially from noradrenaline to values lower than 0.30 µg/kg/min before starting the vasopressin dose reduction.

Hemodynamic variables measured by invasive monitoring showed that there was minimal variation in cardiac output (CO) values at all times: T1 = 7.48 L/min, T2 = 7.66 L/min, T3 = 7 56 L/min, T4 = 6.62 L/min, T5 = 6.42 L/min and T6 = 6.24 L/min, recording an average of 6.99 L/min. Pulmonary extravascular water index presented the following absolute values: T1 = 12.48 mL/kg, T2 = 12.16 mL/kg, T3 = 20.72 mL/kg, T4 = 9.74 mL/kg, T5 = 9.58 mL/kg and T6 = 9.32 mL/kg, with an average of 12.33 mL/kg. The pulmonary vascular permeability index indicated a linear decreasing behavior: T1 = 2.88, T2 = 2.74, T3 = 2.68, T4 = 2.3, T5 = 2.2 and T6 = 2.2, with an average of 2.5.

The average lactate variation was 3.30 mmol/L, 2.40 mmol/L, 2.25 mmol/L, 1.87 mmol/L and 1.67 mmol/L from T1 to T5, respectively. After 24 hours of MB suspension (T6), lactate increased from 1.67 to 2.06 mmol/L and norepinephrine returned to 0.03 µg/kg/min, demonstrating a benefit of MB in the control hemodynamic (Fig. 3).

Using MB did not cause any serious adverse events in this case series.

**Figure 4.**
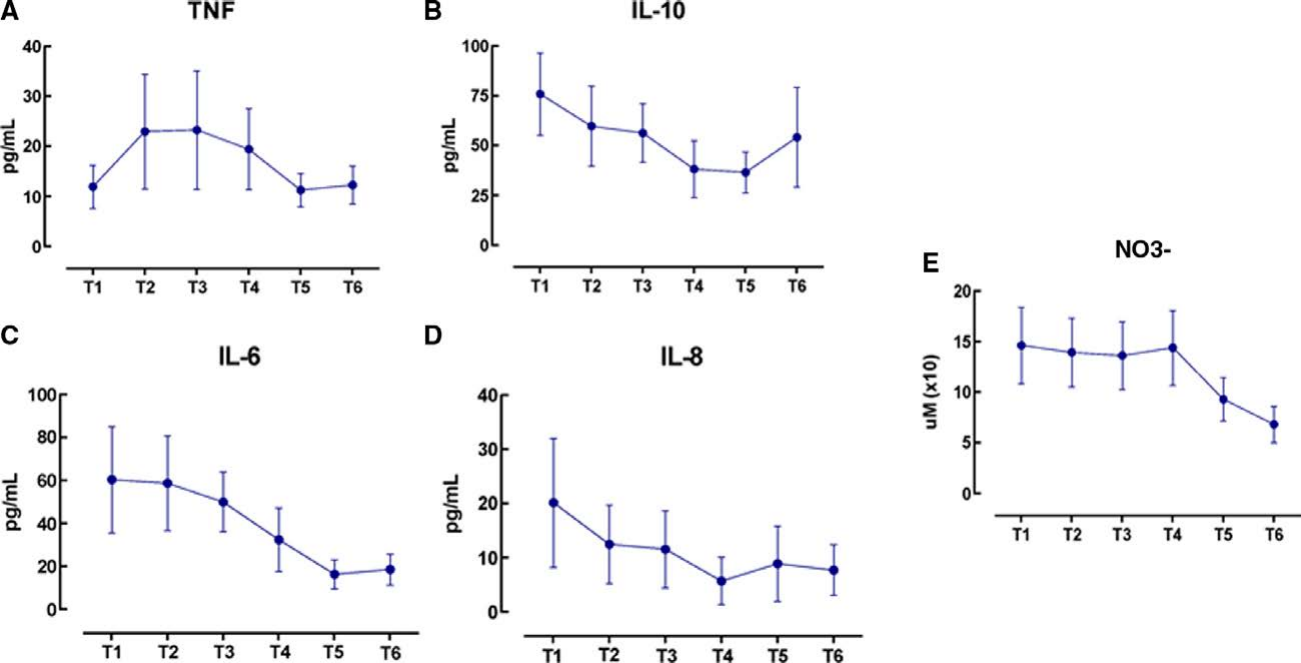
Serum dosage of (A) IL-10, (B) TNF-α, (C) IL-6 and (D) IL-8 and (E) NO3 in the 6 study times. (A) We observed a gradual decrease in serum IL-10 levels being more pronounced in T4, but in T6 there was an increase, which may be related to the suspension of MB before this measurement. (B) There was a tendency to decrease TNF-α over time. (C and D) The IL-6 and IL-8 cytokines initially increased with progressive and marked reduction in the times after the onset of MB, demonstrating a possible role of MB in reducing the inflammatory activity of these patients. (E) Serum levels of NO3 at the 6 study times. We observed a sharp drop in serum NO3 dosage starting in the first 24 hours. IL = interleukin, MB = methylene blue, NO = nitric oxide, NO3 = serum nitrate, TNF-alfa = tumor necrosis factor.

When evaluating the concentration of NO3, we observed high values up to T4, the cause of the inflammatory activity present in septic shock. At T5, there was a sharp drop that was maintained until T6, with a tendency to stabilize to values within normal limits (Fig. 4).

When evaluating IL-10, an anti-inflammatory cytokine, we observed a gradual decrease, being more pronounced at T4, but at T6, there was an increase (Fig. 4).

Regarding the dosage of TNF-α, an inflammatory cytokine, we observed an opposite behavior from that of IL-10 from T1 to T2, where there was an increase in this cytokine. From T2 to T3, there was a decrease that was maintained at T4 and a sharper drop from T4 to T5, rising again at T6 (Fig. 4).

When we evaluated the ILs IL-6 and IL-8, inflammatory cytokines, we observed elevated levels on T1 with a progressive decrease along all other times (Fig. 4).

## 3. Discussion

Septic shock is a lethal disease responsible for a large proportion of ICU deaths, even with currently applied therapies focused on fluid resuscitation, the use of vasopressors and early empirical antibiotic therapy. Considering the multifactorial pathophysiology of septic shock and the mechanism of action of vasopressors, some patients may not respond adequately, which can lead to maintenance of vasodilation, hypotension and morbidity and mortality.

MB may be a good adjunctive strategy associated with first-choice vasopressors in the treatment of septic shock.^[9]^ The medical literature does not present robust studies on the use of BM in septic shock, however, even with a small sample, the results are promising regarding the association of BM with the treatment of these patients. Theoretically, inhibition of excessively produced NO could act favorably by preventing systemic vasodilation and reducing microvascular injury in septic individuals. MB, a nonselective inhibitor of soluble guanylate cyclase and NO synthase, has been studied in endotoxemic animals and patients with septic shock by decreasing plasma levels of the stable metabolites of NO, nitrites and nitrates, as well as cGMP.^[17–19]^

A recent literature review included 15 studies with 832 patients including septic shock, vasoplegic syndrome, and ischemia-reperfusion injury. MB and vasopressors were administered concomitantly, demonstrating improvement in hemodynamics, decreased need for vasopressors, reduced lactate levels and improved survival in patients with vasodilatory shock. However, the study included patients with vasodilation secondary not only to septic shock.^[20]^

In this series of cases, we used the MB in the early phase of the septic shock to reverse vasodilation, which is the main determinant of hemodynamic instability in septic shock. We emphasize the importance of using MB in the early phase of the septic condition, as guanylate cyclase stocks still exist. Its use in rescue situations shows brief effects with a rapid return to hypotension.

Several uncontrolled investigations in patients with septic shock have showed restoration of MAP with bolus administration or short-term infusion of MB. Some studies used MB to reverse vasoplegia in the postoperative period of major surgeries with good results.^[21–25]^ Other case reports used MB to treat patients with anaphylactic shock, refractory shock in poly traumatized patients and in the postoperative period of liver transplantation.^[26–28]^ Other studies have indicated the benefit in reducing vasopressors and maintaining adequate levels of MAP in septic shock,^[5,6,10,29]^ however these are small studies without robust evidence for this approach.

Using MB has also been described in 2 randomized controlled trials, which evaluated its use in the treatment of hypotension secondary to septic shock, with the potential for reduction and early weaning of vasopressors. The first is a pilot study of a randomized, controlled, unblinded clinical trial that included patients with septic shock within the first 24 hours, randomized 1:1 to receive MB (n = 10) or isotonic saline solution (n = 10), adjuvant to conventional treatment. Compared with the control group, MB reduced the need for norepinephrine, epinephrine, and dopamine by up to 87%, 81%, and 40%, respectively. MB also reduced body temperature and plasma nitrate/nitrite concentration. Five patients treated with MB survived, versus 3 patients who received conventional treatment.^[6]^ However, this study did not demonstrate a major impact on the outcome.

The second study was a randomized, double-blind, placebo-controlled clinical trial where patients received MB 0.5 mg/kg/h (n = 15) or a similar volume of isotonic saline (n = 15) intravenously for 6 hours. The main objective was to evaluate the plasma concentrations of TNF-α, IL-1, IL-2, IL-6 and IL-8. MB administration had no significant effect on plasma cytokine levels, blood gases and biochemical parameters. MB administration resulted in increased MAP and methemoglobin levels. The study concluded that MB infusion did not change cytokine levels or outcome in the population studied.^[7]^ However, this study did not perform invasive hemodynamic monitoring to show more accurate micro and macro hemodynamic variables.

Another study investigated short-term intravenous administration of MB in patients with septic shock, demonstrating an increase in MAP and systemic vascular resistance, but not increased CO, oxygen delivery, or oxygen consumption. There was also no sustained clinical improvement and the mortality rate remained high. The authors concluded that MB administration resulted in increased vascular tone and myocardial function, however the study was limited to a short-term infusion of MB. Suggesting that longer administration of MB deserves further investigation.^[30]^

In this series of cases, the standard treatment added to MB showed a reduction in lactate levels, associated with a reduction in using vasopressors, with their suspension up to 48 hours after the beginning of the association with MB. After 24 hours of suspension of MB, there was an increase in the serum lactate dosage from 1.67 to 2.06 mmol/L (value at the upper limit of normality) and a return of noradrenaline to 0.03 µg/kg/min (dose below the recommended minimum, which is 0.05 µg/kg/min), these effects can be attributed to the inhibition of excessive NO/cGMP generation, restoration of vascular sensitivity to catecholamines, which may suggest that the continuous infusion of MB was contributing to better control hemodynamics and tissue perfusion.

Hemodynamic variables measured by invasive monitoring showed minimal variation in CO values at all times, probably MB may have contributed to the maintenance of myocardial function seen in septic shock.^[6]^ The pulmonary extravascular water index showed high values at all times, the initial inflammatory condition can explain this behavior, since the values were high and showed a clear decrease after T3. MB may have had an immunomodulatory effect. The pulmonary vascular permeability index showed values within the normal range at all times, without worsening during treatment.

When we evaluated the serum levels of NO3, we observed high values up to T4, which can probably be attributed to the inflammatory activity present in septic shock. The sharp drop that occurs in T5 may suggest the effect of MB through the inhibition of soluble guanylate cyclase and NO synthase. This result is supported by the literature.^[19]^

IL-10 showed a gradual decrease, being more accentuated at T4, but at T6 there was an increase that may be related to the suspension of MB. Regarding the TNF-α dosage, we observed a behavior opposite to that of IL-10 from T1 to T2. When we evaluated the ILs IL-6 and IL-8, we observed high levels in T1 with a progressive decrease over all other times, demonstrating a reduction in inflammatory activity with the use of MB in these patients. The literature has few robust studies evaluating the dosage of cytokines in patients who used MB in septic shock. As mentioned above, Memis et al^[7]^ evaluated the dosage of TNF-α, IL-1, IL-2, IL-6 and IL-8 and found no changes in serum levels during the use of MB. We speculate these findings differ from our study, as the MB infusion was performed for only 6 hours, probably the longer use may provide a maintenance of this effect.

Our findings show a reduction in the dose of vasopressors during the administration of MB, which is also demonstrated in case reports and small studies, however the invasive hemodynamic monitoring using the EV1000 platform has not yet appeared in the literature for this type of study. This monitoring associated with the dosage of inflammatory, anti-inflammatory and NO3 mediators allows us a better access to the hemodynamic variables that can change during the approach to septic shock. In addition, we can more reliably infer the action of MB in the body of patients with septic shock.

Based on the above, it is clear the great relevance of studying new adjuvant treatments for septic shock to optimize the existing therapeutic arsenal and enable new alternatives in the future.

## 4. Conclusion

This case series suggests that MB used early in the treatment of septic shock associated with standard care may be useful in reducing vasopressor dose, inflammatory cytokines and lactate levels. Further studies are still required to further validate these findings. We are currently carrying out a randomized clinical trial to validate this findings (Clinical registration: https://ensaiosclinicos.gov.br/rg/RBR-96584w4).

## Author contributions

**Formal analysis:** Mayra Gonçalves Menegueti.

**Investigation:** Fabio Luis-Silva, Lucas Sato, Leandro Moreira Peres, Corina dos Reis Sepeda, Bruno C. Petroski-Moraes, Mariana Dermínio Donadel, Gabriela Bortoleto Gallo, Maria Cecília Jordani, Fabiola Mestriner, Christiane Becari, Maria Auxiliadora-Martins.

**Supervision:** Mayra Gonçalves Menegueti, Maria Auxiliadora-Martins.

**Validation:** Maria Auxiliadora-Martins.

**Writing – review & editing:** Anibal Basile-Filho, Paulo R. B. Evora, Olindo Assis Martins-Filho, Maria Auxiliadora-Martins.
